# Analysis of tear inflammatory molecules and clinical correlations in evaporative dry eye disease caused by meibomian gland dysfunction

**DOI:** 10.1007/s10792-020-01489-z

**Published:** 2020-06-29

**Authors:** Xingdi Wu, Xiang Chen, Yajuan Ma, Xueqi Lin, Xuewen Yu, Suhong He, Chenqi Luo, Wen Xu

**Affiliations:** 1 Eye Center, Affiliated Second Hospital, School of Medicine, Zhejiang University, 88 Jiefang Road, Hangzhou, 310009 China; 2 Zhejiang Rongjun Hospital, Jiaxing, China; 3 Suichang Hospital of Traditional Chinese Medicine, Suichang, China

**Keywords:** Dry eye disease, Meibomian gland dysfunction, Inflammatory molecule, Clinical feature

## Abstract

**Purpose:**

To compare the levels of inflammatory molecules in tear samples between patients with meibomian gland dysfunction (MGD)-related evaporative dry eye (EDE) and healthy subjects and to analyze the correlations between the levels of tear inflammatory molecules and ocular surface parameters.

**Methods:**

A total of 30 MGD-related EDE patients (48 eyes) and ten healthy volunteers (15 eyes) were enrolled. Dry eye-related examinations and questionnaires were obtained from all participants. The levels of nine inflammatory molecules were determined through multiplex bead analysis.

**Results:**

Inflammatory molecules including ICAM-1, IFN-γ, CXCL8/IL-8, IL-6, TNF-α and IL-12p70 were detected in 100% of the patients, while IL-1α, IL-1β and IL-10 were detected in 56.25%, 13.60% and 45.83% of the patients, respectively. Moreover, ICAM-1, IL-8, IL-6, TNF-α, IL-12p70 and IFN-γ were detected in 86.67–100% of the healthy subjects, and the detection rates of IL-10, IL-1α and IL-1β were below 50%. The levels of IL-8, IL-6, IFN-γ and ICAM-1 were significantly higher in the patient group compared with the control group. In addition, IL-8 and IL-6 were negatively correlated with Schirmer I test. Besides, IFN-γ was negatively correlated with tear film breakup time. Furthermore, ICAM-1 and IL-6 were positively correlated with meibography score.

**Conclusions:**

Collectively, patients with MGD-related EDE had higher levels of inflammatory molecules in their tears, and some molecules were correlated with ocular surface parameters. These findings suggested that inflammation played an important role in MGD-related EDE, and several inflammatory molecules could be used in the diagnosis and the treatment of MGD-related EDE.

## Introduction

Dry eye disease (DED) is a most commonly diagnosed eye dysfunction in ophthalmology [1, 2]. It is defined as a multifactorial disease of tears and ocular surface that results in symptoms of discomfort, visual disturbance and tear film instability with potential damage to the ocular surface [3, 4]. The DED can mainly be classified into aqueous tear-deficient dry eye (ADDE) and evaporative dry eye (EDE) [5]. Sjögren’s syndrome (SS) is the most typical cause of ADDE, whereas meibomian gland dysfunction (MGD) is the most common cause of EDE [6–8]. The clinical features of these two different classes are specific in the moderate forms of the disease, while they become superimposable in severe forms [9, 10].

Recent studies have demonstrated that inflammation plays an important role in the pathogenesis of DED [11–14]. Studies have shown increased levels of interleukin (IL)-1, IL-6, IL-8 and tumor necrosis factor (TNF)-α in the tear film and conjunctival epithelium of DED patients. Moreover, some of them are associated with the severity of the disease and correlated with various parameters of tear film and ocular surface [15–19]. There are other immunopathological changes, such as immune activation and upregulation of adhesion molecules, including HLA-DR and intercellular cell adhesion molecule-1 (ICAM-1) [20, 21]. According to the inflammatory reaction of the ocular surface, several anti-inflammatory therapies have been used clinically, such as topical corticosteroids, cyclosporine A and lifitegrast [22, 23].

In the present study, we investigated and compared the levels of IL-1α, IL-1β, TNF-α, IL-10, chemokine (C-X-C motif) ligand 8 (CXCL8)/IL-8, IL-6, IL-12p70, interferon (IFN)-γ and ICAM-1 in tear samples between MGD-related EDE patients and normal controls. Moreover, we also analyzed their correlations with disease severity and ocular surface parameters. Our findings provided the evidence that these inflammatory molecules could be potential supplements for the diagnostic and therapeutic regimens for MGD-related EDE.

## Methods

### Patients

A total of 30 MGD-related EDE patients (15 males, 15 females; mean age ± standard deviation [SD] of 53.6 ± 14.85 years, range 18–81 years) consisting of 48 eyes were enrolled in the present study. In addition, ten healthy volunteers (four males, six females; mean age ± SD of 26.33 ± 1.72 years, range 24–30 years) consisting of 15 eyes were recruited as the control group. This prospective study was approved by the Institutional Review Board, Second Affiliated Hospital, Medical College of Zhejiang University, Hangzhou, China. All participants voluntarily signed the informed consent after full explanation of this study. All patients were diagnosed with MGD-caused EDE. Biomicroscopic examination was carried out on the lid margins and meibomian glands, and tear film breakup time (TBUT), Schirmer I test, Ocular Surface Disease Index (OSDI) and dry eye infrared examination, including inferior tear meniscus height, bulbar redness and meibography, were also examined in all participants. Diagnosis criteria of EDE included a score of OSDI > 20, TBUT ≤ 10 s, Schirmer I test score < 10 mm/5 min and meniscus height ≤ 0.35 mm. MGD was diagnosed according to the International Workshop on MGD: (a) meibomian gland dropout, (b) altered meibomian gland secretion and (c) changes in lid morphology [24]. Inclusion criteria were set as follows: all patients had dry eye-related symptoms for at least 6 months, and only used preservative-free artificial tears (topical anti-inflammatory drugs, such as 0.05% cyclosporine A or steroids) were not used. Exclusion criteria included a history of ocular surgery, contact lens use or ocular therapies other than artificial tears within the last 3 months. Patients were also excluded if they had current pregnancy, nursing, lactation or any systemic diseases, such as diabetes, heart diseases and psychosis. The healthy volunteers were healthy, had no history of ocular disease or systemic disease and did not wear contact lens. The results of examinations were all within normal range.

### Clinical examination

Clinical evaluations were performed following the sequence shown below. Clinical evaluations and collection of tear samples were always performed by the same person in the case of bias.

#### Tear sample collection

Tear collection was performed before any other tests. To collect tear samples, 200 μL of normal saline (NS) was instilled into the inferior fornix (without topical anesthetics). More than 100 μL of tear fluid and NS was collected with a micropipette at the lateral canthus. The tear samples were collected as soon as possible to reduce the stimulation of ocular surface. The fluid was placed into a 200-μL Eppendorf tube and kept on dry ice during examination. Then, the samples were stored at − 80 °C prior to further analyses to avoid repeated freezing and thawing.

#### Biomicroscopic examination

Biomicroscopic examination included evaluations of lid margin signs and meibomian gland secretion. Lid margin signs were assessed as follows: irregular lid margin, vascular engorgement, plugged orifices and displacement of mucocutaneous junction [25]. Meibum quality and expressibility (upper eyelid) were scored as 0 = clear, easily expressed; 1 = cloudy, mild pressure; 2 = cloudy, > moderate pressure; 3 = meibum not expressed, with hard pressure [24].

#### OSDI

OSDI was used to assess the symptoms of ocular irritation related to dry eye and their effects on visual functioning. The 12 questions were subscaled into three categories as follows: vision-related function (six questions), ocular symptoms (three questions) and environmental triggers (three questions). The 12 items of the OSDI questionnaire were graded on a scale of 0–4, where 0 indicates none of the time; 1 represents some of the time; 2 reflects half of the time; 3 indicates most of the time; and 4 represents all of the time. The total OSDI score was then calculated based on the following formula: OSDI = [(sum of scores for all questions answered) × 100]/[(total number of questions answered) × 4] [26].

#### Schirmer I test

The Schirmer I test [27] was performed by placing one sterile strip (Schirmer Tear Test Strips, 5 × 35 mm; Liaoning Meizilin Pharmaceutical Co., Ltd., Liaoning, China) in the lateral canthus of the inferior lid margin of both eyes without topical anesthetics. Subjects were asked to maintain their eyes closed during the test, and the length of wetting was measured in millimeters after 5 min.

#### TBUT

Fluorescein strips (Fluorescein paper; Liaoning Meizilin Pharmaceutical Co., Ltd., Liaoning, China) previously wetted with NS were gently applied to the inferior fornix. Subjects were asked to blink several times. Subsequently, they were asked first to close and then open their eyes. The time between the opening of the eyes and the appearance of the first dry spot was measured three times, and the mean was recorded [5].

#### Dry eye infrared examination

Dry eye infrared examination consisting of measurements of inferior tear meniscus height, bulbar redness and meibography was carried out by using a newly developed corneal topographer (Oculus) as previously described [28, 29]. Meibography scores, which quantitate obstruction of the meibomian glands, were obtained using the following grades for each eyelid: 0 (no loss of meibomian glands); 1 (meibomian gland loss less than one third of the total meibomian gland area); 2 (area loss between one-third and two-thirds of the total meibomian gland area); and 3 (area loss more than two-thirds of the total meibomian gland area). The total meibography score was the sum of the scores of the upper and lower lids and recorded as 0–6.

### Detection of tear inflammatory molecules

Cytokines were analyzed using two Luminex commercial assays with Bio-Plex 200™ System (Bio-Rad, Hercules, California, USA). The concentrations of cytokines (IL-1α, IL-1β, TNF-α, IL-10, IL-6, IL12p70 and IFN-γ) and chemokine (CXCL8/IL-8) were determined with an eight-plex assay (Magnetic Luminex Performance Assay, R&D Systems, Minneapolis, MN, USA). The concentration of ICAM-1 was determined with an ICAM-1 single-plex assay (Magnetic Luminex assay, R&D Systems, Minnesota, USA). A total of 50 μL tear sample was required for each assay following the manufacturer’s protocols. Data were recorded and analyzed with the Bio-plex Data Pro (Bio-Rad, Hercules, California, USA).

### Statistical analysis

Statistics were analyzed using the SPSS for Mac 25.0 (SPSS Inc., Chicago, IL, USA). Data were presented as means ± SD. The data of inflammatory molecules were logarithm-transformed for normal distribution. Inflammatory molecule concentrations in the two study groups (MGD-related EDE group and control group) were compared by the nonparametric Mann–Whitney *U* test. Correlations between clinical parameters and tear inflammatory molecule levels were analyzed using Spearman correlations. P less than 0.05 was considered as statistically significant.

## Results

### Clinical features and inflammatory molecules

The clinical features of the MGD-related EDE group were corresponding to diagnostic criteria (Table 1), while the clinical features of the control group were within normal range. All the MGD patients had the typical eyelid morphology changes, and their meibomian gland assessment values were ≥ 1.

**Table 1:** Demographics and clinical features for MGD-related EDE patients and controls

Parameter	MGD-related EDE	Control
Number of eyes (OD/OS)	48 (21/27)	15 (9/6)
Age (range)	53.6 ± 14.85 (18–81)	26.33 ± 1.72 (25–30)
TBUT (s)	3.54 ± 1.64	8.2 ± 2.68
Schirmer I test (mm)	5.80 ± 2.94	13.13 ± 7.78
OSDI (0–100)	49.16 ± 25.12	20 ± 14.66
Bulbar redness	1.49 ± 0.37	0.93 ± 0.26
Meniscus height (mm)	0.2 ± 0.11	0.32 ± 0.06
Meibography score (0–6)	2.56 ± 0.85	1.67 ± 0.62

*OSDI* Ocular Surface Disease Index (grade estimated by the Oxford scheme), *TBUT* tear breakup time, *MGD* meibomian gland dysfunction, *EDE* evaporative dry eye. Data are presented as means ± SD

Table 2 shows the mean values of inflammatory molecules in tear samples of the two groups and the detection rate. The nine inflammatory molecules were determined in tear samples of all participants. In the MGD-related EDE group, ICAM-1, IFN-γ, IL-8, IL-6, TNF-α and IL-12p70 were detected in all patients. IL-1α was detected in 56.25% of samples. The detection rates of IL-1β and IL-10 were below 50%. Moreover, the detection rates of ICAM-1, IL-8, IL-1β, IL-1α, IL-6, IL-10, TNF-α, IL12p70 and IFN-γ in the control group were 86.67%, 100%, 13.30%, 26.67%, 86.67%, 33.33%, 100%, 86.67% and 100%, respectively (Table 2).

**Table 2:** Levels of inflammatory molecules in MGD-related EDE group and control group

Cytokine (ng/mL)	MGD-related EDE	Detection rate (%)	Control	Detection rate (%)
ICAM-1	2,140.41 ± 1009.94	100.00	1,394.51 ± 344.39	86.67
IL-8	59.39.74 ± 51.27	100.00	22.03 ± 16.67	100.00
IL-1β	1.69 ± 1.16	12.50	1.04 ± 1.32	13.30
IL-1α	2.75 ± 1.78	56.25	4.74 ± 4.33	26.67
IL-6	6.11 ± 4.89	100.00	2.64 ± 2.67	86.67
IL-10	12.74 ± 16.05	45.83	7.12 ± 3.01	33.33
TNF-α	5.65 ± 3.40	100.00	4.22 ± 0.52	100.00
IL-12p70	2.77 ± 1.87	100.00	2.5 ± 1.45	86.67
IFN-γ	4.79 ± 4.44	100.00	2.51 ± 0.99	100.00

*IFN* interferon, *IL* interleukin, *TNF* tumor necrosis factor, *ICAM* intercellular cell adhesion molecule, *MGD* meibomian gland dysfunction, *EDE* evaporative dry eye

**Fig. 1:**
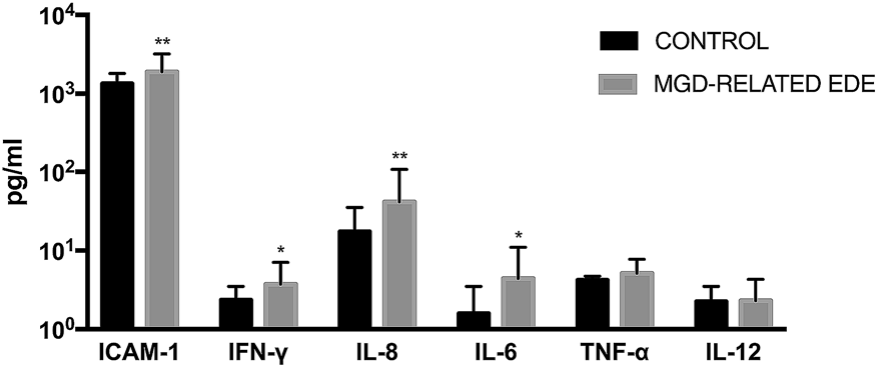
Tear inflammatory molecule levels in MGD-related EDE group and control group. Comparison of tear inflammatory molecule mean values between MGD-related EDE group and control group. Data are presented on a logarithmic scale, and bars represent values. P values were calculated by the nonparametric Mann–Whitney *U* test (*P < 0.05; **P < 0.01

We analyzed the difference of inflammatory molecules between the MGD-related EDE group and control group. The data for the nine molecules detected in more than 50% of samples were analyzed (Fig. 1). The levels of TNF-α, IL-8, IL-6, IFN-γ, IL-12p70 and ICAM-1 were higher in the MGD-related EDE group compared with the control group. However, not all of these changes were statistically significant. ICAM-1 (*P* = 0.002), IFN-α (*P* = 0.012), IL-8 (*P* = 0.003) and IL-6 (*P* = 0.014) levels were significantly increased in the MGD-related EDE group compared with the control group. TNF-α (*P* = 0.222) was increased in the MGD-related EDE group, but there was no statistical difference. IL-12p70 (*P* = 0.978) level was barely changed between the two groups.

### Correlation between clinical features and inflammatory molecules

**Fig. 2:**
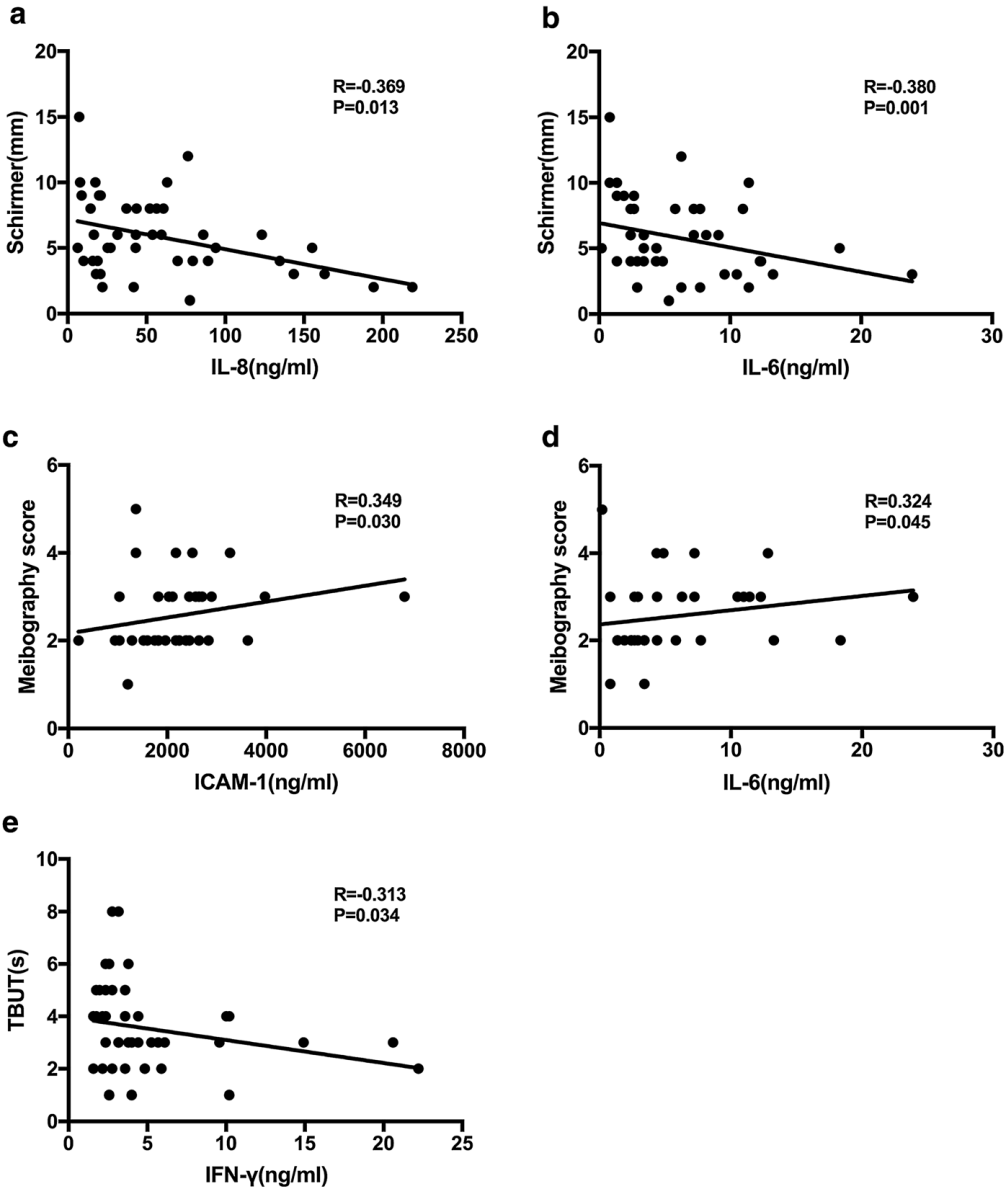
Correlation between inflammatory molecule levels in tear samples and clinical parameters in MGD-related EDE patients. IL-8 and IL-6 were significantly correlated with Schirmer I test. IFN-γ was significantly correlated with TBUT. ICAM-1 and IL-6 were significantly correlated with meibography score. Spearman correlation test was used to analyze correlations

We performed correlation analyses between inflammatory molecules and ocular surface parameters for the seven molecules detected in more than 50% of MGD-related EDE patients (Table 2), including ICAM-1, IFN-γ, IL-8, IL-6, TNF-α, IL-12p70 and IL-1α. Only IL-8, IL-6, ICAM-1 and IFN-γ were statistically correlated with ocular surface parameters. In the MGD-related EDE group, the levels of IL-8 and IL-6 were negatively correlated with Schirmer I test. Moreover, ICAM-1 and IL-6 were positively correlated with meibography score. In addition, TBUT showed negative correlation with IFN-γ (Fig. 2). Meniscus height and the OSDI score were not significantly correlated with inflammatory molecules (Table 3).

**Table 3:** Correlation between tear inflammatory molecules and clinical parameters in MGD-related EDE patients

Inflammatory molecules	Meniscus height	Schirmer I test	TBUT	Bulbar Redness	Meibography score	OSDI
ICAM-1						
*r*	− 0.203	− 0.215	− 0.131	0.199	**0.349**	0.069
*P* value	0.166	0.156	0.385	0.266	**0.03**	0.64
IFN-γ						
*r*	− 0.085	− 0.139	− **0.313**	0.142	0.246	− 0.183
*P* value	0.565	0.362	**0.034**	0.429	0.131	0.213
IL-8						
*r*	− 0.171	− **0.369**	− 0.106	0.3	0.233	− 0.222
*P* value	0.244	**0.013**	0.483	0.09	0.153	0.129
IL-1α						
*r*	− 0.057	− 0.07	− 0.161	0.203	0.115	− 0.34
*P* value	0.776	0.739	0.421	0.405	0.609	0.082
IL-6						
*r*	− 0.092	− **0.38**	0.041	0.227	**0.323**	− 0.051
*P* value	0.533	**0.01**	0.786	0.203	**0.045**	0.729
TNF-α						
*r*	0.018	− 0.241	− 0.277	0.246	0.238	− 0.097
*P* value	0.902	0.11	0.062	0.167	0.145	0.513
IL-12						
*r*	− 0.14	− 0.183	0.119	0.017	0.142	− 0.244
*P* value	0.344	0.229	0.432	0.925	0.387	0.095

*IFN* interferon, *IL* interleukin, *TNF* tumor necrosis factor, *ICAM* intercellular cell adhesion molecule, *OSDI* Ocular Surface Disease Index (grade estimated by the Oxford scheme), *TBUT* tear breakup time, *MGD* meibomian gland dysfunction, *EDE* evaporative dry eye. Spearman correlation coefficient (*r*) was used to analyze the associations between variables

Significant correlations are in bold font

## Discussion

DED is regarded as a multifactorial disorder, of which the etiology and pathological mechanism are complicated. Recently, many studies have shown that DED is an inflammatory disease and also has many features similar to autoimmune disease, indicating that inflammation plays a significant role in the progression and symptoms of DED [30, 31]. In our daily life, the eyes are continually exposed to desiccating stress. Nevertheless, such condition is counteracted by homeostatic mechanisms which regulate tear secretion and distribution in response to signals from the ocular surface [4]. In DED, this homeostatic balance is always disturbed, adversely affecting tear film stability and osmolarity. This is recognized as the beginning of the inflammatory events and surface damage [32].

MGD is a condition of a chronic, diffuse abnormality of the meibomian glands [33], and it is the most common cause of EDE [34]. Many investigators have reported that DED can lead to elevated levels of IL-1, IL-6, IL-8, IFN-γ and TNF-α in the conjunctival epithelium and tears [15–17, 35]. However, most of the studies have focused on ADDE patients with SS. Only very few studies have investigated the cytokine levels in tears of patients with MGD-related EDE. In the present study, we investigated several inflammatory molecules in tear samples of MGD-related EDE patients. Moreover, we also explored the correlations between clinical parameters of MGD-related EDE patients and the levels of inflammatory molecules in tears.

The production of proinflammatory cytokines (IL-1, IL-6, IFN-γ and TNF-α), which mediate intercellular communication, is increased by osmotic, inflammatory, and mechanical damages [31]. According to our study, IFN-γ and TNF-α were detected at higher concentrations in the MGD-related EDE group, which was consistent with the previously published reports [13]. Although the difference of TNF-α was not statistically significant, there was still an upward trend. Previous studies have shown that desiccating stress stimulates production of inflammatory mediators, such as IL-1β and TNF-α, in the ocular surface epithelium by activating mitogen-activated protein kinase (MAPK) pathway [36]. The high expression of TNF-α initiates a series of inflammatory responses on the ocular surface, which may lead to the occurrence and development of DED. Furthermore, some researchers recommend TNF-α blockers which can improve DED through effectively ameliorating corneal inflammation and suppressing other cytokines [37, 38]. In terms of IFN-γ, some basic studies have found that there is a recruitment of T cells in the conjunctiva using dry eye models of humans and animals. The increase in Th1 cytokine IFN-γ in tears indicated that IFN-γ-producing inflammatory cells might be recruited to the ocular surface in EDE. IFN-γ has also been associated with the decrease in goblet cells [39], which may lead to dysfunction of the tear film. This could explain the observation that IFN-γ was inversely correlated with TBUT in this study. IL-6 is considered to be one of the most important molecules in DED. This molecule is significantly increased in the tears of DED patients, and it is correlated with various ocular surface parameters [13, 18]. Moreover, it is also regarded as a main characteristic for the immune response in human microbial keratitis and pediatric lacrimal duct obstruction [40]. In our present study, the type of MGD-related EDE could be divided into both moderate and severe forms. Previous study has shown that the IL-6 level is not increased in moderate EDE [12]. However, we found that the IL-6 level was significantly increased in MGD-related EDE patients. We also found that IL-6 was significantly correlated with meibography score and Schirmer I test. These results indicated that the IL-6 expression was not increased in moderate EDE, while it was increased with the development of EDE. The level of IL-6 might be used as an indicator to define the disease severity and evaluate the anti-inflammatory drug efficacy for DED. IL-1α was detected in 56.25% patients, while it was only detected in 26.67% subjects in the control group. Additionally, the detection rates of IL-1β and IL-10 were less than 50% in both groups. In the present study, some patients with mild clinical signs might have lower levels of these cytokines. Moreover, 200 μL NS might also dilute the concentrations of these inflammatory molecules, leading to the lower detection rates of these cytokines.

IL-8 is a potent proinflammatory cytokine that has been shown to be chemotactic for neutrophils, lymphocytes and basophils [41]. The chemokine IL-8 was significantly increased in the tears of MGD-related EDE patients compared with the control group in this study. The amplification of IL-8 is a dramatic mechanism in DED through infiltration and activation of T lymphocytes, which may lead to damage to the lacrimal gland and ocular surface tissue through cytotoxicity and apoptosis [42]. We also found that IL-8 was negatively correlated with Schirmer I test. Therefore, high level of IL-8 could be a typical sign of MGD-related EDE.

As a common ocular disorder, DED is associated with autoimmune inflammation of the lacrimal gland and ocular surface [30, 31]. Cell adhesion molecules are cell surface proteins that facilitate cellular migration and promote the infiltration of immune cells into the ocular surface of DED patients. ICAM-1 is one of the first discovered adhesion molecules of immunoglobulin superfamily, which is consistently identified in the conjunctiva and lacrimal glands in DED patients [43, 44]. In this study, we investigated the ICAM-1 level in the tear samples of MGD-related EDE patients and its correlation with ocular surface parameters. We found that the ICAM-1 level, same as IFN-γ, was significantly increased in MGD-related EDE tear samples. The expression of ICAM-l is always increased by inflammatory cytokines, such as IL-1β, TNF-α and IFN-γ [45]. We could infer that the increased expression of ICAM-1 was associated with IFN-γ. The upregulation of ICAM-1 is essential for homing and activation of T cells and cytokine release of the ocular surface, which leads to an inflammatory milieu, resulting in symptoms of eye discomfort [46]. In the past few years, lifitegrast, an LFA-1 antagonist that blocks interaction of ICAM-1 to LFA-1, has been approved by the US Food and Drug Administration (FDA) for the treatment of DED, which is the first drug showing improvements in both symptoms and signs of DED.

As a questionnaire, OSDI provides a assessment of the symptoms subjectively. Due to the lack of association between signs and symptoms in DED patients [47], it is difficult to identify the severity of DED with a single examination, which may explain why we did not find the correlation between inflammatory molecules and OSDI in our study. Moreover, only nine inflammatory molecules were analyzed in this study, and some relevant molecules might be neglected. Therefore, more inflammatory molecules and more clinical parameters are needed to be investigated in further studies. In addition, there are other limitations in this study, such as relatively small sample size and large age difference between two groups.

## Conclusions

Collectively, we found that several inflammatory molecules were increased in tear samples of MGD-related EDE patients compared with the healthy controls. Moreover, some of them were also correlated with clinical features. These results suggested that the changes in expressions of inflammatory molecules were associated with ocular surface alteration in MGD-related EDE disease. In other words, the inflammatory molecules were associated with the severity of MGD-related EDE. Our findings provided further evidence of the pathogenesis of DED, especially MGD-related EDE, which could be potential supplements for the diagnostic and therapeutic regimens of MGD-elated EDE.
